# Supplementary material for: Identification of Dihydromyricetin and Metabolites in Serum and Brain Associated with Acute Anti-Ethanol Intoxicating Effects in Mice
Source: Int J Mol Sci. 2021 Jul 12;22(14):7460. doi: 10.3390/ijms22147460 (PMC8307506; doi:10.3390/ijms22147460)
Supplement: Supplementary file 1 [file ijms-22-07460-s001.zip › ijms-1279704-supplementary.pdf]

Supplementary Data

Contents: A. MRM mass transitions and retention times of developed UPLC-QqQ-MS/MS method for analysis of dihydromyricetin and metabolites in biological samples. **B**. Chromatogram of serum sample **C**. Experimental procedures and characterization for the preparation of compounds 3′-O-methyl-dihydromyricetin (3′-Me-DHM), 4′-O-methyl-dihydromyricetin (4′-Me-DHM) and 4′-dehydroxy-dihydromyricetin (4′-DeOH-DHM).

A. MRM mass transitions and retention times of developed UPLC-QqQ-MS/MS method for analysis of dihydromyricetin and metabolites in serum and brain samples.

Compound	Metabolic Route	Quantifier Transition 1	Qualifier Transition 2	Qualifier Transition 3	Retention Time (min)
DHM	-	319→125	319→193	319→125	4.23
4-OH-DHM	Reduction	321→125	321→195	-	ND
3′-DeOH-DHM 4′-DeOH-DHM	Dehydroxylation	303→125	303→109	321→193	5.32
5-DeOH-DHM 7-DeHO-DHM	Dehydroxylation	303→125	303→109	321→193	ND
5-Me-DHM 7-Me-DHM	Methylation	333→139	333→125	333→207	5.60, 5.65
3′-Me-DHM 4′-Me-DHM	Methylation	333→125	333→139	333→193	5.27, 5.37
Sulfate-DHM	Sulfation	399→193	-	-	ND
G-DHM	Glucuronidation	495→319	-	-	2.12
G-Me-DHM	Methylation + Glucuronidation	509→333	-	-	3.45, 3.55
G-DeOH-DHM	Glucuronidation + Dehydroxylation	479→303	-	-	3.52
G-4-OH-DHM	Glucuronidation +Reduction	497→321	-	-	ND

ND; not detected.

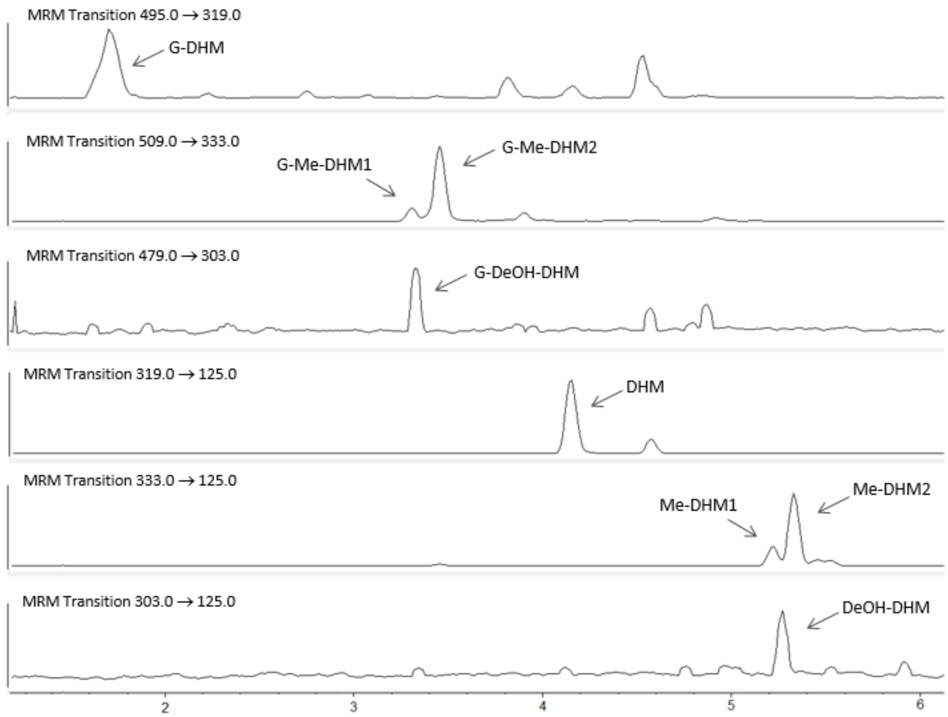

B. Representative UPLC-QqQ/MS chromatograms of serum sample collected from male mouse 15 min post IP administration of 50 mg/kg dihydromyricetin).

C. Experimental procedures and characterization for the preparation of compounds 3′-O-methyl-dihydromyricetin (3′-Me-DHM), 4′-O-methyl-dihydromyricetin (4′-Me-DHM), 4′-dehydroxy-dihydromyricetin (4′-DeOH-DHM).

1] 1-(2,4,6-tris(methoxymethoxy)phenyl)ethan-1-one, While stirring on ice, 6.3 mmols of 1-(2,4,6-trihydroxyphenyl)ethan-1-one was added to 30 mL of DMF and 4.5 equiv. of NaH was slowly added under nitrogen. Next, over 15 min, 4.5 equiv. of chloromethoxymethyl ether and the reaction stirred for 12 h, warming to rt. The reaction was then quenched with cold water and extracted 2 times with Et_2O and dried over Na_2SO_4. Product was isolated with silica gel flash column, with a solvent gradient of 10-25% EtOAc/hexane, to yield 3.9 mmols 1 (62% yield). ^{1}H-NMR (500 MHz, DMSO-d_6) δ 2.4 (3H, s), 3.4 (9H, s), 5.2 (6 H, s) 6.5 (2H, s) ^{13}C-NMR δ (500 MHz, DMSO-d_6) 32.8, 56.3, 94.7, 97.1, 116.9, 154.8, 159.2, 200.7.

2] Benzaldehyde, 3-methoxy-4,5-bis(phenylmethoxy)-, 6.3 mmols of 3,4-dihydroxy-5-methoxybenzaldehyde was added to 25 mL of DMF with 2.1 equ. of K_2CO_3 and stirred at room temperature. After 15 min, equ. BnBr. At 48 hrs, another 1 equ. BnBr and stirred for 24 h, at no starting material was observed by LCMS. After confirmation of product on LC-MS, two extractions with ether and water, followed by a silica gel column with 5-20% EtOAc in hexane to yield 6.2 mmols 2 (98% yield). ^{1}H-NMR δ 3.9 (s, 3H), 5.1 (s, 2H), 5.2 (s, 2H), 7.30-7.5 (m, 10H), 9.9 (s, 1H) ^{13}C-NMR δ 56.6, 70.5, 74.6, 107.5, 108.5, 127.1, 128.4, 128.5, 132.2, 137.8, 142.5, 153.0, 154.2, 192.3.

3] Benzaldehyde, 4-methoxy-3,5-bis(phenylmethoxy)-, 6.6 mmols of 3,4,5-trihydroxybenzaldehyde was added to 40 mL of DMF with 1.2 equ. of K_2CO_3 and stirred at 85 °C for 1 h. After reaction was cooled on ice, 1 equ. of methyl iodide was added and the reaction stirred for 24 h at rt. In the same flask, 3.3 equ. 1M BnBr and an additional 2.4 equ. K_2CO_3 were added and stirred for 24 hrs at room rt. After confirmation of product on LC-MS, two extractions with ether and water, followed by a silica gel column with 0-15%

EtOAc in hexane to yield 4.6 mmols 3 (72% yield). ^{1}H-NMR δ 3.3 (s, 3H), 5.7 (s, 4H), 7.1 (s, 2H), 7.4-7.8 (m, 10H) ^{13}C-NMR δ 66.2, 79.5, 113.6, 121.0, 127.1, 129.0, 138.9, 139.0, 147.4, 154.4, 191.0.

4](E)-3-(3-methoxy-4,5-bis(methoxymethoxy)phenyl)-1-(2,4,6-tris(methoxymethoxy)phenyl)prop-2-en-1-one, 1.2 mmols of 1 (1 equ.) with 5 equ. NaOH in 50 mL 1:1 THF/EtOH and stirred for 15 min at rt. Next, 1 equi. of 2 was added and reaction stirred for 12 hrs at room temperature. The product was isolated with two EtOAc extractions and subsequent filtration of crystals formed in EtOAc, to yield 1.0 mmols 5 (86% yield). ^{1}H-NMR δ 3.2 (s, 9H), 3.8 (s, 3H) 5.0 (s, 4H), 5.2 (s, 6H), 7.1 (s, 2H), 7.17 (s, 2H), 7.19 (m, 2H), 7.3 (m, 4H), 7.4 (m, 4H) ^{13}C-NMR δ 56.3, 56.4, 70.6, 74.6, 94.5, 97.1, 107.8, 115.0, 126.9, 128.1, 128.9, 130.5, 137.5, 139.3, 145.4, 152.7, 153.9, 155.9, 159.2, 193.8.

5] (E)-3-(3,5-bis(benzyloxy)-4-methoxyphenyl)-1-(2,4,6-tris(methoxymethoxy)phenyl)prop-2-en-1-one, 2.9 mmols of 1 (1 equ.) with 5 equ. NaOH in 50 mL 1:1 THF/EtOH and stirred for 15 min at rt. Next, 2.9 equ. of benzaldehyde, 3,5-bis(phenylmethoxy) was added and reaction stirred for 12 hrs at room temperature. The product was isolated with two EtOAc extractions and subsequent filtration of crystals formed in EtOAc/Hexanes, yielding 2.4 mmols 6 (85% yield). ^{1}H-NMR δ 3.4 (s, 9H) 3.8 (s, 3H), 5.3 (s, 4H), 6.24 (s, 6H), 6.48 (s, 2H), 6.75 (s, 2H), 7.2-7.5 (m, 10H) ^{13}C-NMR δ 56.2, 64.3, 73.6, 94.4, 94.7, 99.8, 104.1, 112.5, 126.4, 128.0, 128.9, 137.5, 140.2, 141.6, 155.5, 161.8, 166.4, 193.2.

6] (E)-3-(3,5-bis(methoxymethoxy)phenyl)-1-(2,4,6-tris(methoxymethoxy)phenyl)prop-2-en-1-one, 0.85 mmols of 1 (1 equ.) with 5 equ. NaOH in 20 mL EtOH and stirred for 15 min at rt. Next, 2.9 equi. of 4 was added and reaction stirred for 12 hrs at room temperature. The product was isolated with two EtOAc extractions and subsequent filtration of crystals formed in EtOAc, yielding 2.4 mmols 6 (77.5% yield) ^{1}H-NMR (500 MHz, DMSO-d_6) δ 3.3 (s, 9H) 5.1 (s, 4H) 5.2 (s, 6H) 6.5 (s, 1H) 6.7 (s, 4H) 7.0 (s, 4H) 7.33-7.41 (m, 10H) ^{13}C-NMR δ 56.4, 69.9, 94.6, 97.1, 107.7, 111.8, 114.8, 128.2, 128.3, 128.8, 133.0, 136.8, 146.8, 155.6, 159.3, 160.2, 193.8.

7] (3-(3-methoxy-4,5-bis(methoxymethoxy)phenyl)oxiran-2-yl)(2,4,6 tris(methoxymethoxy)phenyl)-methanone, 0.96 mmols 5 was dissolved in 20 mL of 1:1 MeOH/Dioxane, to which 2 equ. of NaOH (1M aq.) and 10 equ. of H_2O_2 (50% w/v aq.) were added and reaction stirred for 16 hrs, when reaction was complete based on LCMS. Product was isolated with two EtOAc extractions and silica gel column chromatography with 5-25% EtOAc/Hexane, to yield 0.95 mmols 8 (99% yield). ^{1}H-NMR (500 MHz, DMSO-d_6) δ 3.3 (s, 9H) 3.8 (s, 3H), 3.88 (s, 1H), 3.93 (s, 1H) 5.2 (s, 3H) 6.5 (s, 6H) 6.7 (s, 2H), 6.8 (s, 2H), 7.30-7.35 (m, 10H) ^{13}C-NMR δ 56.3, 57.6, 60.8, 70.6, 74.5, 76.5, 94.6, 94.8, 97.8, 102.9, 128.03, 128.5, 128.8, 131.8, 137.4, 138.1, 153.9, 156.5, 160.1, 162.2, 192.3.

8] (3-(3,5-bis(benzyloxy)-4-methoxymethoxy)oxiran-2-yl)(2,4,6-tris(methoxymethoxy)phenyl)-methanone, 1.4 mmols 6 was dissolved in 20 mL of 1:1 MeOH/Dioxane, to which 2 equ. of NaOH (1M aq.) and 10 equ. of H_2O_2 (50% *w/v* aq.) were added and reaction stirred for 16 hrs, when reaction was complete based on LCMS. Product was isolated with two EtOAc extractions and silica gel column chromatography with 5-25% EtOAc/Hexane, to yield 1.1 mmols 9 (80% yield). ^{1}H-NMR (500 MHz, DMSO-d_6) δ 3.4 (s, 9H), 3.7 (s, 3H), 3. (s, 1H), 4.13 (s, 1H), 5.1 (s, 4H), 5.2 (s, 6H), 6.5 (s, 2H), 6.8 (s, 2H), 7.28-7.40 (m, 10H). 56.6, 60.8, 71.6, 74.5, 76.5, 94.6, 95.8, 103.1, 112.1, 127.2, 127.5, 128.8, 137.4, 138.1, 153.9, 153.2, 160.5, 162.2, 192.3.

9] (3-(3,5-bis(methoxymethoxy)phenyl)oxiran-2-yl)(2,4,6-tris(methoxymethoxy)phenyl)methanone, 0.37 mmols 7 was dissolved in 20 mL of THF, to which 3 equiv. of diethylamine and 10 equ. of H_2O_2 (50% *w/v* aq.) were added and reaction stirred for 16 hrs, when reaction was complete based on LCMS. Product was isolated with two EtOAc extractions and silica gel column chromatography with 5–25% EtOAc/Hexane, to yield 0.24 mmols 10 (64.9% yield). ^{1}H-NMR (500 MHz, DMSO-d_6) δ 3.3 (s, 9H), 3.9 (s,

1H), 4.1 (s, 1H), 5.12 (s, 4H), 5.14 (s, 6H), 6.5 (s, 2H), 6.63(s, 1H) 6.7 (s, 2H), 7.33-7.42 (m, 10H) ^{13}C-NMR (500 MHz, DMSO-d_6) δ 56.3, 56.4, 69.9, 74.8, 94.5, 94.6, 97.1, 107.7, 114.8, 128.1, 128.9, 129.9, 136.8, 137.4, 155.6, 159.3, 160.2, 193.8.

3′-Me-DHM] 2-(3,4-dihydroxy-5-methoxyphenyl)-3,5,7-trihydroxychroman-4-one (3′-Me-DHM), 0.27 mmols of 8 was added to 35 mL of 1:4 THF:MeOH and 5 mL of 11.6M HCl. Reaction was stirred for 12 hrs at rt, at which time the reaction was complete by LCMS. Product was extracted 2 times with EtOAc, and rotovaped to dryness. Subsequent benzyl deprotection was carried out on crude product in 10 mL MeOH, a small scoop of 10% Pd/C, and stirred for 48 hrs at rt. Product was isolated with 2 EtOAc extractions, followed by C18 flash column with 0-15% H2O/ACN with 0.1% FA, to yield 4.7 mg 3′-Me-DHM (yield = 4.8%). ^{1}H-NMR (500 MHz, DMSO-d_6) δ 3.8 (s, 3H), 5.3 (s, 1H), 5.7 (s, 1H), 5.8 (s, 1H), 5.85 (s, 1H), 5.86 (s, 1H), 6.5 (s, 2H), 8.4 (s, 1H), 9.0 (s, 1H), 10.2 (s, 1H), 10.9 (s, 1H). ^{13}C-NMR (500 MHz, DMSO-d_6) δ 58.2, 72.4, 82.3, 95.9, 97.3, 101.2, 105.0, 107.9, 126.1, 130.3, 145.9, 147.9, 162.3, 163.8, 165.4, 196.9.

4′-Me-DHM] 2-(3,5-dihydroxy-4-methoxyphenyl)-3,5,7-trihydroxychroman-4-one (4′-Me-DHM), 0.7 mmols of 9 was added to 35 mL of 1:4 THF:MeOH and 5 mL of 11.6M HCl. Reaction was stirred for 12 h at rt, at which time the reaction was complete by LCMS. Product was extracted 2 times with EtOAc, and rotovaped to dryness. Subsequent benzyl deprotection was carried out on 117 mg product in 10 mL MeOH, a small scoop of 10% Pd/C, and stirred for 48 hrs at rt. Product was isolated with 2 EtOAc extractions, followed by C18 flash column with 0-15% H2O/ACN with 0.1% FA, to yield 4.1 mg 4′-Me-DHM (yield = 5.6%). ^{1}H-NMR (500 MHz, DMSO-d_6) δ 3.7 (s, 3H), 4.4 (s, 1H), 5.1 (s, 1H), 5.3 (s, 1H), 5.9 (s, 1H), 6.6 (s, 2H), 7.1 (s, 1H), 9.3 (s, 2H), 11.2 (s, 1H), 12.9 (s, 1H). ^{13}C-NMR (500 MHz, DMSO-d_6) δ 52.8, 68.6, 83.4, 95.3, 95.4, 106.2, 107.0, 109.3, 135.1, 138.4, 150.6, 164.0, 165.6, 191.1.

4′-DeOH-DHM] 2-(3,5-dihydroxyphenyl)-3,5,7-trihydroxychroman-4-one (4′-DeOH-DHM), 0.12 mmols of 10 was added to 35 mL of 1:4 THF:MeOH and 5 mL of 11.6M HCl. Reaction was stirred for 12 h at rt, at which time the reaction was complete by LCMS. Product was extracted 2 times with EtOAc and dried under vacuum. Subsequent benzyl deprotection was carried out on 117 mg product in 10 mL MeOH, a small scoop of 10% Pd/C, and stirred for 48 hrs at rt. Product was isolated with 2 EtOAc extractions, followed by C18 flash column with 0-15% H2O/ACN with 0.1% FA, to yield 10.5 mg 3′-DeOH-DHM (yield = 28%). ^{1}H-NMR (500 MHz, DMSO-d_6) δ 5.8 (s, 1H), 5.8 (s, 1H), 5.89 (s, 1H), 5.92 (s, 1H), 6.2 (s, 1H), 6.28 (s, 1H), 6.31(s, 1H), 9.3 (s, 2H), 10.8 (s, 1H), 11.9 (s, 1H) ^{13}C-NMR (500 MHz, DMSO-d_6) δ 72.1, 83.9, 94.3, 95.5, 100.9, 105.0, 106.4, 139.5, 158.6, 162.8, 163.8, 167.3, 197.8.
